# Biodegradability of PLA-Based Nonwoven Fabrics with Poultry Feathers

**DOI:** 10.3390/polym17070957

**Published:** 2025-03-31

**Authors:** Jagoda Jóźwik-Pruska, Krystyna Wrześniewska-Tosik, Tomasz Kowalewski, Justyna Wietecha, Michalina Pałczyńska, Magdalena Szalczyńska

**Affiliations:** Lukasiewicz Research Network—Lodz Institute of Technology, Skłodowskiej-Curie Str. 19/27, 90-570 Łódź, Poland; krystyna.wrzesniewska-tosik@lit.lukasiewicz.gov.pl (K.W.-T.); tomasz.kowalewski@lit.lukasiewicz.gov.pl (T.K.); justyna.wietecha@lit.lukasiewicz.gov.pl (J.W.); michalina.palczynska@lit.lukasiewicz.gov.pl (M.P.); magdalena.szalczynska@lit.lukasiewicz.gov.pl (M.S.)

**Keywords:** nonwoven, biodegradation, keratin, feathers, poultry waste, circular economy

## Abstract

Geotextiles are widely used for separation, drainage, filtration, and erosion control, as well as for enhancing plant growth conditions. The objective of this study was to evaluate the impact of incorporating poultry feathers on the biodegradation rate of nonwoven geotextiles in arable soil. The research was conducted under laboratory conditions, with biodegradation assessed based on mass loss. The findings confirmed that the presence of keratin-rich waste positively influenced the biodegradation rate of the tested materials. Additionally, the potential ecotoxicological effects of biodegradation were examined, revealing no adverse impact on microbiological activity. Statistical analysis demonstrated a correlation between material composition and biodegradation time. This study represents a significant step toward the sustainable management of poultry feather waste in agricultural applications. The tested materials could serve as an environmentally viable alternative for long-term applications, aligning with ecological sustainability principles by simultaneously enriching soil with essential nutrients and promoting waste valorization.

## 1. Introduction

The Circular Economy (CE) approach presents a viable solution to the challenges associated with economic growth, particularly the increasing production and accumulation of waste. The poultry industry has experienced a substantial expansion in production scale, leading to the generation of significant quantities of poultry by-products over the years [1]. The disposal of poultry feathers poses considerable challenges due to the high volume of waste produced, resulting in substantial environmental burdens and waste management costs [2]. Currently, only a small fraction of this waste is utilized industrially, including applications in textile production, insulation, biodegradable polymer synthesis, and microbiological culture media. To mitigate the environmental impact of poultry feather waste, it is imperative to develop sustainable and cost-effective management strategies [3].

Chicken feathers consist of approximately 90% keratin, 1% lipids, and 8% water. Keratin is naturally insoluble due to the presence of peptide bonds, making it resistant to degradation [4]. However, it represents a valuable source of organic matter that can contribute to soil fertility and productivity enhancement. Consequently, chicken feathers have been identified as a potential raw material for the production of nonwoven geotextiles due to their low cost, lightweight nature, high availability, and durability. Research indicates that geotextiles influence key soil parameters such as bulk density, porosity, and water-holding capacity, all of which are critical for maintaining soil quality and protection. Some studies suggest that the incorporation of natural geotextiles into soil can enhance nutrient availability [5].

Geotextiles are durable, flexible materials designed to facilitate fluid flow through their structure [6]. They can be manufactured from both biodegradable and non-biodegradable materials, with natural fibers being particularly suitable due to their high strength, moisture retention capacity, and low elasticity [7]. When introduced into the soil, natural geotextiles contribute to improved soil structure and microbial activity [8]. During biodegradation, they release organic matter and nutrients, fostering optimal conditions for plant growth. Natural geotextiles have diverse applications, including crop protection, erosion control, and soil reinforcement in embankment stabilization.

A key advantage of biodegradable nonwoven geotextiles is their ability to decompose naturally in the soil after their functional lifespan, eliminating the need for post-use collection. The degradation products serve as valuable nutrients for plants, further enhancing soil fertility and promoting sustainable agricultural practices.

Poultry feathers (PF) consist of more than 90% keratin, a fibrous and insoluble structural protein. Keratin exhibits significant resistance to physical, chemical, and biological degradation, including enzymatic hydrolysis by proteolytic enzymes. This resilience is primarily attributed to its molecular structure, particularly the presence of disulfide bridges, which reinforce its stability [9]. As a result, the natural biodegradation of keratin in the environment is slow and inefficient. However, mechanical pretreatment of feathers enhances the accessibility of keratin to soil microorganisms, thereby facilitating the biodegradation process.

For agrotextiles derived from poultry feathers to be effectively utilized in improving soil trophicity, the presence of keratinolytic microorganisms capable of degrading keratin is essential. These microorganisms are the subject of extensive biotechnological and microbiological research [10,11,12]. The soil environment serves as a natural habitat for many of these organisms, which utilize keratin as a source of nitrogen and carbon. Through enzymatic activity, they produce keratinolytic enzymes that catalyze the breakdown of keratin into bioavailable compounds. Among these microorganisms, bacteria of the genus Bacillus have been identified as particularly efficient in keratin degradation [13,14].

Polymer degradation is a complex process that involves three key stages: biodeterioration, biofragmentation, and assimilation. The initial phase, biodeterioration, occurs when microorganisms colonize the polymer surface, leading to alterations in its mechanical, chemical, and physical properties [15,16]. In the subsequent phase, biofragmentation, the polymer chains are cleaved into oligomers and monomers, rendering them more soluble. The final and most critical stage is assimilation, during which microorganisms metabolize the degraded polymer components, resulting in the production of carbon dioxide (CO_2_), energy, and biomass under aerobic conditions, or carbon dioxide (CO_2_) and methane (CH_4_) under anaerobic conditions [17].

This study evaluated the biodegradability of nonwoven fabrics under controlled laboratory conditions, with a particular focus on structural changes occurring in the materials during degradation. The analysis considered both the composition of the nonwoven fabrics and the degree of feather fragmentation to determine their impact on the biodegradation process.

## 2. Materials and Methods

### 2.1. Materials

The test material was nonwovens manufactured by the needle-punching method at the Łukasiewicz Research Network—Łódź Institute of Technology. Four types of nonwovens were selected for testing:

- 100% PLA—PLA_0.1, PLA_0.2—differing in basis weight,
- 50% PLA/50% cotton—PLA.C_01, PLA.C_0.2—differing in basis weight,
- PLA with feathers added—PLA_1—PLA_4—differing in feather content and basis weight,
- PLA/cotton (1:1) with feather addition—PLA.C_1—PLA.C_5—differing in feather content and basis weight.

The following raw materials were used to manufacture the nonwovens:

- shredded chicken feathers (producer: CEDROB S.A. Z.D., Niepołomice, Poland),
- PLA—staple fibres TREVIRA^®^ 400 6.7 dtex bright rd 60 mm, (producer: Trevira GmbH, Bobingen, Germany)
- cotton fibres, length 20–30 mm, diameter 20–30 µm (waste from the production of cosmetic pads).

### 2.2. Methods

Biodegradability was assessed using an accredited and validated method conducted on a laboratory scale. The tests were performed on samples produced on a quarter-technical scale. Each 5 × 5 cm sample was tested in triplicate under repeatability and reproducibility conditions. The final result was determined as the arithmetic mean of three repetitions, with measurement uncertainty components taken into account. As a reference material, 100% cotton was used. According to existing standards, biodegradable materials should achieve 90% decomposition within a maximum period of 24 weeks.

Given the potential application of the tested materials, soil was selected as the biodegradation medium. The experiments were conducted at a temperature of 30 ± 2 °C and humidity levels ranging from 60% to 75%. The test soil consisted of unfertilized universal soil with a total microbial count of no less than 10⁶ CFU. Samples were placed in research reactors filled with the test soil and stored in a controlled heat chamber, which maintained the required environmental parameters (temperature and humidity). The decomposition process was monitored over a period of up to six months, with daily soil moisture measurements. The experiment was carried out under aerobic conditions.

Ecotoxicity assessments were performed to evaluate the impact of microbiological degradation of the test samples on the total microbial population in the test matrix. These assessments followed an accredited and validated research procedure, “Assessment of the Influence of Natural and Synthetic Materials on Soil Microflora”, developed in accordance with international standards (EN ISO 11133 and EN ISO 11133:2014-07/A1; EN ISO 4833-1:2013-12; EN ISO 19036:2020-04). Sampling intervals were aligned with intermediate evaluations of the biodegradation process.

Due to the complexity and heterogeneity of the nonwoven materials, it was not possible to precisely describe the reaction stoichiometry. The study included an analysis of the carbon-to-nitrogen (C/N) ratio in the test medium throughout the biodegradation process, as this ratio remains relatively stable in soil organic matter.

Statistical analyses were conducted using the STATISTICA software package (StatSoft, Kraków, Poland, version 9.0). Correlation and cluster analyses were performed.

Microscopic analysis, including elemental composition assessment, was conducted using a Phenom ProX G6 Desktop Scanning Electron Microscope (SEM) equipped with an Energy Dispersive Spectroscopy (EDS) detector (Thermo Fisher Scientific, Waltham, MA, USA). The analysis was carried out in Backscattered Electron Detection (BSD) mode at an operating voltage of 15 kV.

## 3. Results

Needle-punched nonwoven fabrics composed of polylactic acid (PLA), cotton, and shredded poultry feathers were subjected to biodegradation under controlled soil conditions. The degree of biodegradation was assessed based on mass loss in the tested nonwoven groups. Variations in biodegradation rates were observed among the different sample groups, directly correlating with the composition of the nonwoven fabrics.

Table 1 presents a summary of the obtained biodegradation results, including the degradation degree and the composition of the tested samples.

**Figure 1:**
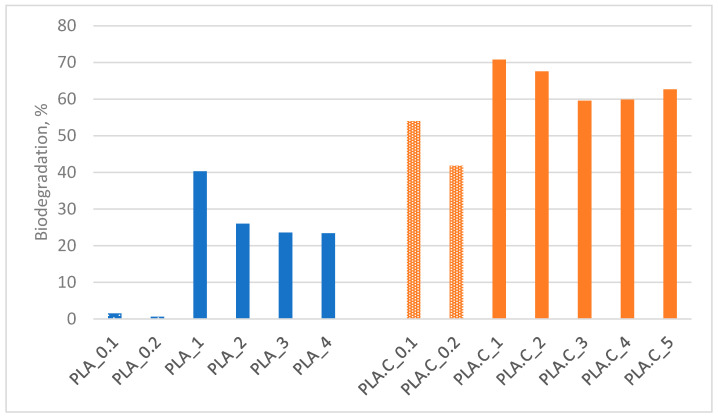
Biodegradation degree of tested nonwovens; PLA—polylactic acid, C—Cotton.

The obtained results are presented in Figure 1 and divided into groups based on composition. Observations show that the degree of biodegradation of the tested materials is similar in percentage to the content of cotton and/or feathers. For samples consisting of 100% PLA, the degree of decomposition was negligible.

**Figure 2:**
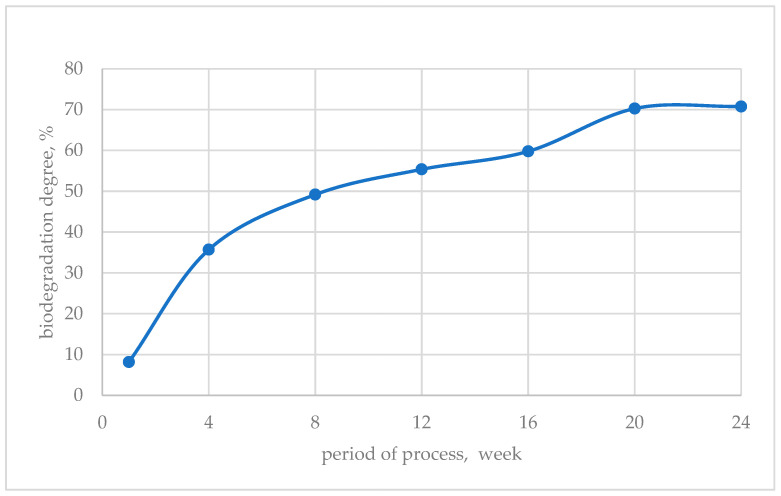
Biodegradation progress for PLA.C_1 sample.

**Figure 3:**
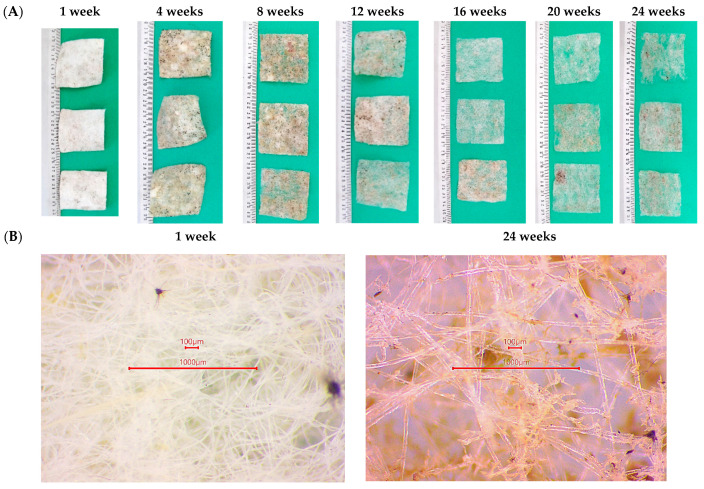
Photographic documentation of biodegradation progress for PLA.C_1 sample; (**A**) macroscopic view, (**B**) microscopic view—optical microscope.

For the sample with the highest degree of biodegradation—PLA.C_1, Figure 2 shows the progress of biodegradation over time. Changes in the structure of the PLA.C_1 nonwoven can also be observed in the images shown in Figure 3—both macroscopic images (A) and under the optical microscope (B).

**Figure 4:**
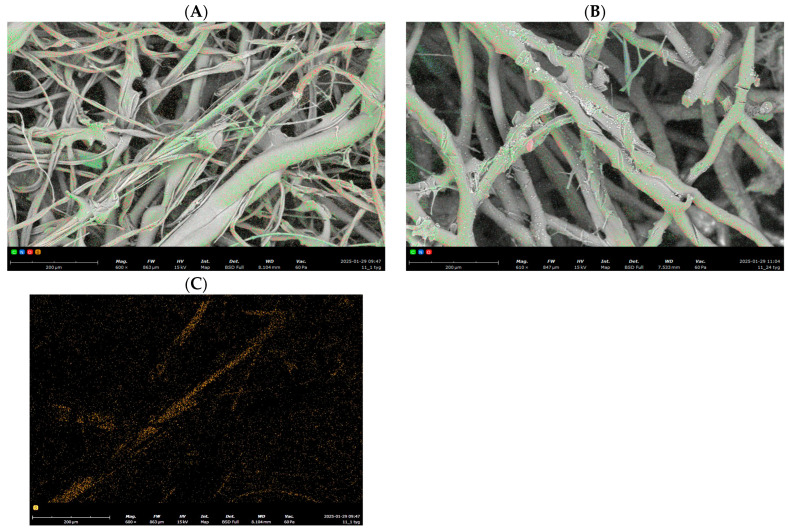
SEM Analysis with EDS of Sample PLA.C_1—Complete map of elemental distribution in the sample after (**A**) 1 week and (**B**) 24 weeks of biodegradation; (**C**) map of sulfur distribution in the sample after 1 week of biodegradation.

For a more detailed examination of the nonwoven fabric structure, scanning electron microscopy (SEM) analysis was performed (Figure 4), accompanied by elemental surface analysis (EDS) for a selected PLA-cotton-feather three-component nonwoven fabric after 1 and 24 weeks of biodegradation.

In Figure 4A, three distinct fiber types are visible: fibers with the largest diameter and a smooth surface, identified as PLA; fibers with a significantly smaller diameter, corresponding to cotton; and irregularly shaped structures, representing feathers. Since feathers contain sulfur, the sulfur distribution map (Figure 4C) clearly indicates the presence of feather components in the initial nonwoven fabric.

After 24 weeks of biodegradation (Figure 4B), the SEM analysis reveals that primarily intact PLA fibers and trace amounts of short cotton fibers remain. The EDS analysis (Table 2) further confirms the absence of sulfur in the final sample, indicating the complete decomposition of the feather component.

**Figure 5:**
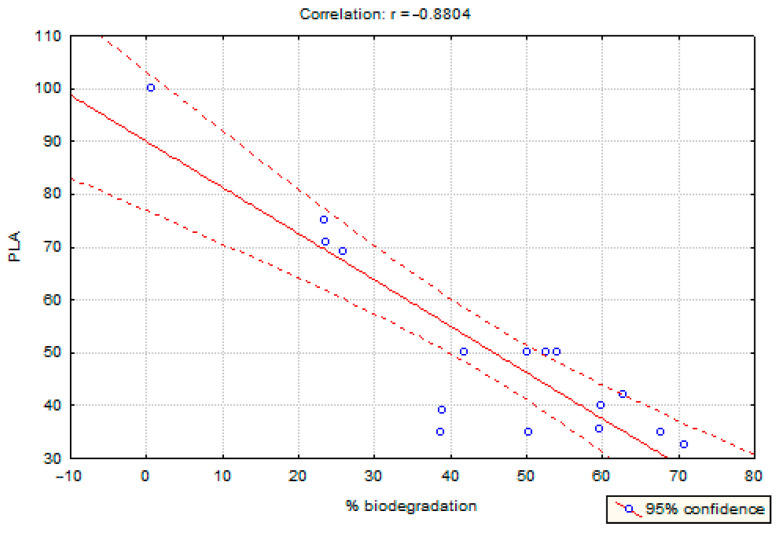
The relationship between PLA content and biodegradability.

**Figure 6:**
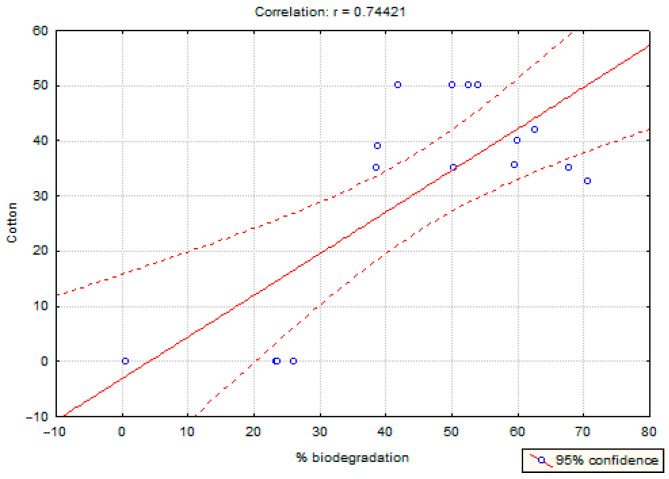
The relationship between cotton content and biodegradability.

Correlations between selected properties: PLA content (−0.88), cotton content (0.74), feather content (0.17), grammage (−0.38), feather fragmentation degree (0.19), and biodegradation degree were examined. A strong correlation was shown for PLA content (reverse correlation) and cotton (positive correlation). Figure 5 and Figure 6 present the described dependencies. The obtained results show that the higher the PLA content, the lower the degree of decomposition, and the more cotton, the higher the degree of decomposition.

**Figure 7:**
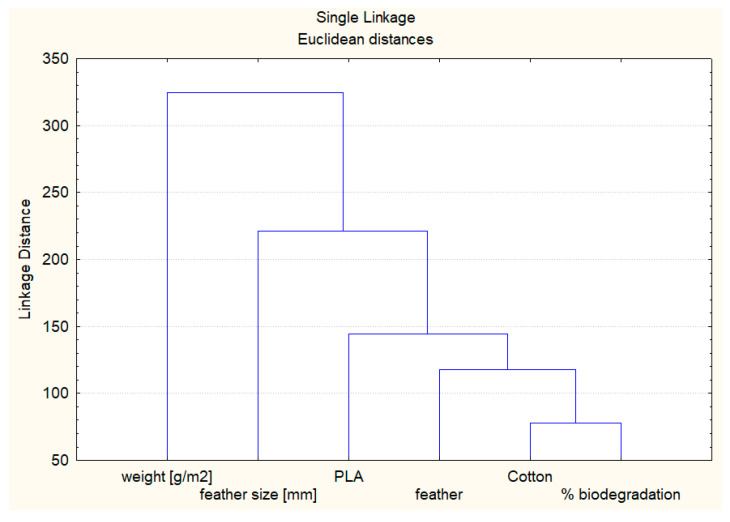
Relationship between the examined features.

In order to illustrate the relationship between the examined features, a cluster analysis was performed (Figure 7). The test organizes items (features) into groups, or clusters, on the basis of how closely associated they are.

Cluster analysis confirmed a strong correlation between the degree of biodegradation and cotton content. The analysis also showed a correlation between this group and feather content.

Given that biodegradation is a natural process mediated by microorganisms, ecotoxicity assessments were conducted to evaluate the impact of material decomposition on soil microbial communities. The tests aimed to determine whether the biodegradation of the nonwoven fabrics influenced the total number of microorganisms responsible for sample degradation.

**Figure 8:**
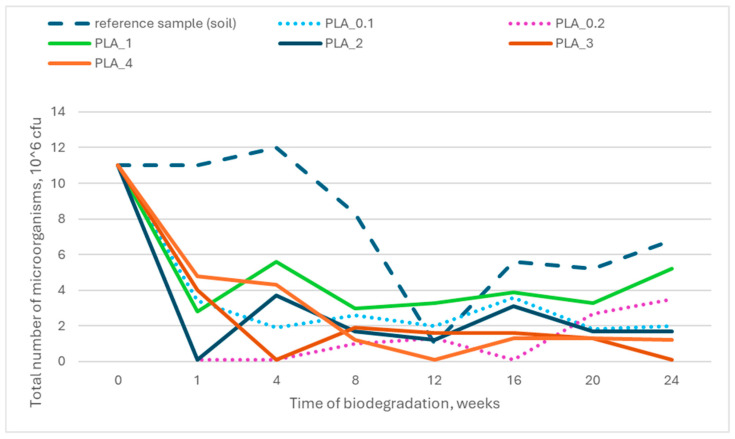
Relationship between total number of microorganisms in soil and time of biodegradation for PLA_x series samples.

**Figure 9:**
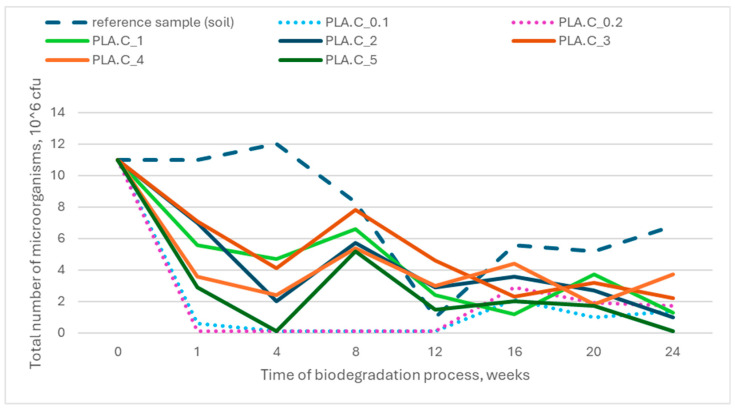
Relationship between total number of microorganisms in soil and time of biodegradation for PLA.C_x series samples.

The results indicated no significant deviations from the reference sample (soil without contact with the tested materials), suggesting that the biodegradation process did not adversely affect microbial populations. Figure 8 and Figure 9 illustrate the total number of microorganisms present in the individual research reactors throughout the biodegradation process.

## 4. Discussion

The application of polylactic acid (PLA) in various fields has been widely documented, primarily due to its advantageous properties, including ease of processing, low toxicity, and high transparency. However, one of its major limitations is its slow biodegradation rate in mesophilic and psychrophilic environments [18].

Cotton fibers are among the most commonly used natural materials. While inherently biodegradable, their decomposition rate can be influenced by chemical modifications. The biodegradation process of cotton is primarily based on the depolymerization of cellulose macromolecules [19]. Previous studies have demonstrated that nonwoven fabrics incorporating poultry feathers have significant potential for hazardous waste management while simultaneously enriching the natural environment [20].

The present study aimed to evaluate the biodegradation of nonwoven fabrics in a soil environment. The tested materials included nonwovens composed of 100% PLA, as well as blends of 50% PLA and 50% cotton, both with and without the addition of poultry feathers. The primary objective was to assess the impact of feather incorporation on the biodegradation process.

The results demonstrated that nonwovens composed entirely of PLA exhibited only 1.5% degradation after 24 weeks of soil incubation. These findings align with data reported by other researchers on PLA biodegradation. Slezak et al. [15] investigated the biodegradation of PLA with polybutylene adipate terephthalate and various additives, as well as a PLA-based polyester blend with a mineral filler, under soil conditions at an average temperature of 9.4 °C. After one year, the degree of decomposition in their study remained below 0.6%. The authors observed surface erosion of the samples and a reduction in tensile strength. Similarly, Rudnik et al. [21] conducted long-term studies on the decomposition of PLA fibers in soil and reported slow degradation rates. Other studies have also indicated minimal or negligible hydrolysis and biodegradation of PLA in natural environments [22]. According to literature data [23], the estimated biodegradation period of PLA in soil is approximately 43 years.

Several factors influence the degradation rate of PLA, including the isomer ratio, temperature, pH, burial time, humidity, oxygen availability, as well as the shape and size of the material. PLA degradation occurs through multiple mechanisms, including hydrolytic, oxidative, thermal, microbiological, enzymatic, chemical, and photodegradation, all of which primarily induce cracks in the main and side chains. Among these, enzymatic and microbiological degradation have attracted the most interest, as they ultimately lead to the breakdown of PLA into CO_2_ and H_2_O [24,25,26]. Since temperature, pH, thermal conditions, and microbial community composition differ between soil and compost, the degradation rate varies across these environments [27,28].

The biodegradation of PLA in soil primarily depends on the presence of water and temperature. The initial stage of the process involves hydrolytic degradation, wherein water penetrates the PLA structure, breaking ester bonds and forming oligo- and monomeric fragments. These water-soluble oligomers then diffuse into the surrounding environment. Surface erosion occurs when the rate of oligomer release exceeds the rate of water diffusion into the sample. Conversely, if water diffusion is faster, degradation occurs throughout the entire volume of the material.

The second stage of PLA biodegradation is driven by microbial activity. Microorganisms decompose the water-soluble units and oligomers into simpler compounds, ultimately leading to the formation of CO_2_ and H_2_O. The rate of PLA degradation is highly dependent on environmental conditions, with higher temperatures and humidity levels accelerating the process [27,29].

In addition to temperature and humidity, factors such as material size and shape also influence the biodegradation rate of PLA [30]. Krawczyk-Walach et al. [31] provided a detailed analysis of the PLA degradation process in soil, emphasizing the role of these variables in determining the overall rate of decomposition.

According [16] to the literature, biodegradation of PLA nonwovens involves three steps:

1. slow decomposition related to the phase of adaptation and colonisation of microorganisms on the surface; hydrolitic degradation which stars with the penetration of water into the structure of nonwoven and leads to the hydrolysis of ester bonds;
2. rapid increase of decomposition which may be related to multiplication of microorganisms; metabolisation of mono- and oligomers dissolved in water into carbon dioxide and water;
3. the third stage may be related with absorbtion of remnants of the polymer (dimers and monomers) by microorganisms as the source of carbon and energy.

Nonwoven fabrics composed of 50% PLA and 50% cotton exhibited approximately 50% biodegradation after 24 weeks. The rate of mass loss was nearly equivalent to the proportion of cotton in the material. These findings suggest that the combination of cotton and PLA does not affect the degradability of PLA. Correlation analysis revealed a significant relationship between material composition and biodegradation rate, with PLA content negatively correlated (−0.88) and cotton content positively correlated (0.74) with biodegradation. This indicates that a higher PLA content reduces the degree of biodegradation, whereas an increased cotton content enhances it.

Furthermore, no significant effect of basis weight on the biodegradation rate was observed. Cluster analysis supported these findings, demonstrating that the highest degree of biodegradation occurred in samples composed of PLA, cotton, and poultry feathers.

For nonwoven fabrics containing feathers, the degree of biodegradation after 24 weeks closely corresponded to the feather content (PLA_1–PLA_4) or the combined content of cotton and feathers (PLA.C_1–PLA.C_5). Analysis of the biodegradation process over time indicates an almost linear degradation pattern in the initial stages, followed by a significant slowdown upon reaching the aforementioned levels. Despite the progression of biodegradation, the remaining sample retained its nonwoven fabric structure, albeit with a reduced basis weight compared to the initial sample.

Microscopic analysis revealed a noticeable loosening of the fabric structure, with PLA fibers remaining largely intact. Since the fiber components in the nonwoven fabric are physically connected without chemical bonding, they degrade independently. Energy-dispersive X-ray spectroscopy (EDS) analysis confirmed the complete removal of feathers from the fabric. Initially, the nonwoven fabric contained approximately 0.8% sulfur by weight, a characteristic element of feathers. However, after 24 weeks, sulfur was no longer detected, indicating the complete decomposition of the feather component. In contrast, the PLA fibers retained their structural integrity, with only fragmented cotton fibers observed in the sample after 24 weeks of biodegradation (Figure 4B).

These findings align with the observations of Südar and Devrim [32], who reported over 90% weight loss for a 100% cotton fabric with a basis weight of approximately 150 g/m^2^ after 16 weeks of soil degradation. The absence of sulfur in the degraded nonwoven fabric further supports the complete biodegradation of the poultry feather component. Vadillo et al. [33] reported that ground feathers in soil degraded by approximately 50% within 12 weeks, with only minor changes occurring thereafter. The variation in biodegradation rates observed in the present study may be attributed to the proportion of cotton, which is more readily colonized by microorganisms. In the initial phase of biodegradation, microbial growth was concentrated on the cotton component, subsequently leading to the degradation of the poultry feather component as microbial colonies expanded.

The feasibility of using PLA for geotextile production has been extensively documented in the literature. Our findings align with these reports, demonstrating the potential for long-term applications of PLA-based nonwoven fabrics. Dharmalingam et al. [34] investigated the biodegradation of PLA nonwovens produced using two techniques: meltblown (MB) and spunbond (SB). Their study assessed tensile strength and crystallinity, revealing that, in the early stages of degradation, microorganisms preferentially consumed more accessible carbon sources, such as amorphous PLA regions. This led to an increase in crystallinity and a corresponding decrease in tensile strength for MB nonwoven fabrics. In contrast, SB nonwovens exhibited only minor physiochemical changes. These results suggest that PLA nonwovens, particularly those produced via the SB method, could be suitable for long-term applications, including row covers and landscape fabrics.

Other studies [35] have explored the potential of blending PLA fibers with natural fibers such as viscose, jute, and hemp in varying proportions. These blends were mechanically formed into webs using a carding and needle-punching technique. Field experiments conducted during winter conditions showed significant structural transformations in the nonwoven mulches, including shrinkage, increased mass per unit area, greater thickness, and reduced air permeability. The proportion of PLA fibers in these blends strongly influenced the structural changes observed. The authors concluded that nonwoven mulches could serve as an alternative to conventional agricultural films.

Miros-Kudra et al. [36] further examined the biodegradation rate of PLA nonwovens produced using the spunbond technique in a composting environment. Their study highlighted the relationship between crystallinity and mechanical strength, indicating that higher crystallinity corresponded to lower tensile strength. The tested nonwovens were reported to fully decompose within 16 weeks under composting conditions, demonstrating their potential for sustainable applications.

Biodegradable or partially biodegradable geotextiles can be employed for soil reinforcement, particularly in applications requiring temporary stabilization or support during vegetation establishment. These materials enhance the immediate stability of soil in areas subjected to light loads, serving as a temporary protective layer while facilitating plant growth, which eventually takes over the stabilizing function [37].

One of the key advantages of biodegradable geotextiles is their ability to provide structural support while allowing plant roots to penetrate through their porous, fibrous structure. Studies have confirmed that feather-based geotextiles, due to their fluffy and permeable nature, enable easier root penetration and establishment [38]. By adjusting the ratio of biodegradable to non-biodegradable fibers in nonwoven fabrics, these materials can be optimized for different soil conditions.

In areas prone to erosion, geotextiles with a higher PLA content may be more effective, as they provide longer-lasting support before degradation. Partial biodegradation facilitates early root development, while the residual nonwoven layer continues to reinforce the substrate, ultimately enhancing soil stabilization over time.

## 5. Conclusions

Sustainable alternatives to conventional plastic mulches in agriculture have gained significant attention. As a result, the development of various types of biodegradable mulches derived from natural sources, such as fibers and biopolymers, has been extensively studied. In this research, nonwoven composites composed of PLA, cotton, and poultry feathers in various quantitative ratios were investigated. The study revealed a strong negative correlation between PLA content and the biodegradation rate, as well as a strong positive correlation between cotton content and biodegradation. The results indicate that a higher PLA content led to lower decomposition, whereas increased cotton content enhanced biodegradation. Additionally, the incorporation of poultry feather waste had a positive effect on the biodegradation process. Microbial analysis further confirmed that the decomposition of the tested materials did not significantly alter the total number of soil microorganisms compared to the reference sample, suggesting no adverse effects on soil microbial balance. The presented materials demonstrate strong potential for long-term applications, such as row covers and landscape fabrics, while exhibiting no adverse environmental impact.

## Figures and Tables

**Table 1:** Composition and biodegradation degree of tested nonwovens.

Nonwoven	Biodegradation Degree, % ± SD	Composition, %			Basic Weight, g/m^2^
		Cotton	PLA	Feather	
PLA_0.1	1.51 ± 0.46	0	100	0	106
PLA_0.2	0.622 ± 0.325	0	100	0	140
PLA_1	40.30 ± 2.73	0	63	37	169
PLA_2	26.0 ± 1.93	0	69	31	202
PLA_3	23.6 ± 5.8	0	71	29	197
PLA_4	23.4 ± 1.74	0	75	25	186
PLA.C_0.1	54.0 ± 1.40	50	50	0	106
PLA.C_0.2	41.8 ± 1.16	50	50	0	122
PLA.C_1	70.8 ± 6.37	32.5	32.5	35	153
PLA.C_2	67.6 ± 2.57	35	35	30	147
PLA.C_3	59.6 ± 2.84	35.5	35.5	29	141
PLA.C_4	59.9 ± 1.23	40	40	20	152
PLA.C_5	62.7 ± 1.21	42	42	16	146

**Table 2:** Elemental content of the PLA.C_1 sample after 1 week and 24 weeks of biodegradation (The table shows colours with which the elements are labelled on the elemental distribution maps—Figure 4).

	Element Number	Element Symbol	Element Name	PLA.C_1, 1 Week	PLA.C_1 24 Weeks
				Weight Conc., %	
	6	C	Carbon	38.900	41.466
	7	N	Nitrogen	3.800	2.711
	8	O	Oxygen	56.500	55.823
	16	S	Sulfur	0.800	-

## Data Availability

Data are contained within the article.

## References

[1] Ibrahim A.D., Rabah A.B., Ibrahim M.L., Magami I.M., Isah J.G., Muzoh O.I. (2014). Bacteriological and chemical properties of soil amended with fermented poultry bird feather. Int. J. Biol. Chem. Sci..

[2] Schmidt W.F. Innovative feather utilization strategies. Proceedings of the National Poultry Waste Management Symposium. Auburn University Printing Services.

[3] Joardar J.C., Rahman M.M. (2018). Poultry feather waste management and effects on plant growth. Int. J. Recycl. Org. Waste Agric..

[4] Lasekan A., Bakar A.F., Hashim D. (2013). Potential of chicken by-products as sources of useful biological resources. Waste Manag..

[5] Pal A., Adhikary R., Bera M., Krishiviswavidyala B.C., Bengal W. (2020). Application of Different Geotextile in Soil to Improve the Soil Health in Humid and Hot Sub Humid Region of West Bengal, India. Int. J. Curr. Microbiol. App. Sci..

[6] Mitchell D.J., Barton A.P., Fullen M.A., Hocking T.J., Zhi W.B., Zheng Y. (2003). Field studies of the effects of jute geotextiles on runoff and erosion in Shropshire, UK. Soil. Use Manag..

[7] Pritchard M., Sarsby R.W., Anand S.C., Horrocks A.R., Anand S.C. (2000). Textiles in civil engineering. Part 2—Natural fibre geotextiles; Horrocks. Handbook of Technical Textiles.

[8] Sarkar T., Perween T., Datta P. (2019). Assessing the Effect of Geotextile Mulch on Yield and Physico-Chemical Qualities of Litchi—A New Technical Approach. Int. J. Curr. Microbiol. Appl. Sci..

[9] Łaba W., Rodziewicz A. (2008). Biodegradation of feather keratin by *Bacillus cereus* in pure culture and compost. EJPAU.

[10] Riffel A., Brandelli A. (2006). Keratinolytic bacteria isolated from feather waste. Braz. J. Microbiol..

[11] Bohacz J., Korniłłowicz-Kowalska T. (2009). Changes in enzymatic activity in composts containing chicken feathers. Bioresour. Technol..

[12] Paul T., Halder S.K., Das A., Bera S., Maity C., Mandal A., Das P.S., Das Mohapatra P.K., Pati B.R., Mondal K.C. (2013). Exploitation of chicken feather waste as a plant growth promoting agent using keratinase producing novel isolate *Paenibacillus woosongensis* TKB2. Biocatal. Agric. Biotechnol..

[13] Brandelli A. (2008). Bacterial Keratinases: Useful Enzymes for Bioprocessing Agroindustrial Wastes and Beyond. Food Bioprocess Technol..

[14] Rodziewicz A., Łaba W., Sobolczyk J., Grzelak A., Drozd J. (2009). Compositing of keratinous waste with bacterial inoculum in a rotary bioreactor. Inż. Ap. Chem..

[15] Slezak R., Krzystek L., Puchalski M., Krucińska I., Sitarski A. (2023). Degradation of bio-based film plastics in soil under natural conditions. Sci. Total Environ..

[16] Emadian S.M., Onay T.T., Demirel B. (2017). Biodegradation of bioplastics in natural environments. Waste Manag..

[17] Briassoulis D., Mistriotis A. (2018). Key parameters in testing biodegradation of bio-based materials in soil. Chemosphere.

[18] Camacho Muñoz R., Villada Castillo H.S., Hoyos Concha J.L., Solanilla Duque J.F. (2024). Aerobic biodegradation of poly(lactic acid) (PLA) in thermoplastic starch (TPS) blends in soil induced by gelatin. Int. Biodeterior. Biodegrad..

[19] Smith S., Ozturk M., Frey M. (2021). Soil biodegradation of cotton fabrics treated with common finishes. Cellulose.

[20] Jóźwik-Pruska J., Wrześniewska-Tosik K., Mik T., Wesołowska E., Kowalewski T., Pałczyńska M., Walisiak D., Szalczyńska M. (2022). Biodegradable nonwovens with poultry feather addition as a 2 way of recycling and waste management. Polymers.

[21] Rudnik E., Briassoulis D. (2011). Degradation behaviour of poly(lactic acid) films and fibres in soil under Mediterranean field conditions and laboratory simulations testing. Ind. Crops Prod..

[22] Chuensangjun C., Pechyen C., Sirisansaneeyakul S. (2013). Degradation Behaviors of Different Blends of Polylactic Acid Buried in Soil. Energy Procedia.

[23] Tsuji H. (2013). Poly(lactic acid). Bio-Based Plastics: Materials and Applications.

[24] Zaaba N.F., Jaafar M. (2020). A review on degradation mechanisms of polylactic acid: Hydrolytic, photodegradative, microbial, and enzymatic degradation. Polym. Eng. Sci..

[25] Tokiwa Y., Calabia B.P. (2006). Biodegradability and biodegradation of poly(lactide). Appl. Microbiol. Biotechnol..

[26] Khabbaz F., Karlsson S., Albertsson A.-C. (2000). PY-GC/MS an effective technique to characterizing of degradation mechanism of poly (L-lactide) in the different environment. J. Appl. Polym. Sci..

[27] Nowak B., Pająk J. (2010). Biodegradacja polilaktydu (PLA). Arch. Gospod. Odpad. I Ochr. Sr..

[28] Karamanlioglu M., Robson G.D. (2013). The influence of biotic and abiotic factors on the rate of degradation of poly(lactic) acid (PLA) coupons buried in compost and soil. Polym. Degrad. Stabil..

[29] Jarerat A., Tokiwa Y. (2003). Poly(L-lactide) degradation by *Saccharothrix waywayandensis*. Biotechnol. Lett..

[30] Artham T., Doble M. (2008). Biodegradation of aliphatic and aromatic polycarbonates. Macromol. Biosci..

[31] Krawczyk-Walach M., Gzyra-Jagieła K., Milczarek A., Jóźwik-Pruska J. (2021). Characterization of Potential Pollutants from Poly(lactic acid) after the Degradation Process in Soil under Simulated Environmental Conditions. AppliedChem.

[32] Sülar V., Devrim G. (2020). Biodegradation Behaviour of Different Textile Fibres: Visual, Morphological, Structural Properties and Soil Analyses. Fibres Text. East. Eur..

[33] Vadillo J., Montes S., Grande H.-J., Verstichel S., Almqvist J., Wrześniewska-Tosik K. (2023). Enhanced Biodegradability in Soil of Chicken Feather by Steam Explosion for Potential Application in Agricultural Biodegradable Plastics. Polymers.

[34] Dharmalingam S., Hayes D.G., Wadsworth L.C., Dunlap R.N. (2015). Analysis of the time course of degradation for fully biobased nonwoven agricultural mulches in compost-enriched soil. Text. Res. J..

[35] Kopitar D., Marasovic P. (2024). Degradation of Biodegradable Nonwoven Mulches in the Winter Period. Polymers.

[36] Miros-Kudra P., Gzyra-Jagieła K., Kudra M. (2021). Physicochemical Assessment of the Biodegradability of Agricultural Nonwovens Made of PLA. Fibres Text. East. Eur..

[37] Mwasha A. (2009). Using environmentally friendly geotextiles for soil reinforcement: A parametric study. Mater. Des..

[38] Kacorzyk P., Strojny J., Białczyk B. (2021). The Impact of Biodegradable Geotextiles on the Effect of Sodding of Difficult Terrain. Sustainability.

